# Evaluation of a Portable Blood Gas Analyzer for Prehospital Triage in Carbon Monoxide Poisoning: Instrument Validation Study

**DOI:** 10.2196/48057

**Published:** 2023-10-06

**Authors:** Matthieu Lyon, Christophe Alain Fehlmann, Marc Augsburger, Thomas Schaller, Catherine Zimmermann-Ivol, Julien Celi, Birgit Andrea Gartner, Nicolas Lorenzon, François Sarasin, Laurent Suppan

**Affiliations:** 1 Division of Emergency Medicine Department of Anesthesiology, Clinical Pharmacology, Intensive Care and Emergency Medicine University of Geneva Hospitals and Faculty of Medicine Geneva Switzerland; 2 School of Epidemiology and Public Health University of Ottawa Ottawa, ON Canada; 3 Unit of Forensic Toxicology and Chemistry University Center of Legal Medicine Lausanne-Geneva Switzerland; 4 Division of Laboratory Medicine Department of Diagnostics Geneva University Hospitals and Faculty of Medicine Geneva Switzerland

**Keywords:** carbon monoxide poisoning, carbon monoxide intoxication, prehospital triage, Avoximeter 4000, CO-oximetry, blood gas, blood work, pulse oximeter, cohort study, carbon monoxide, poisoning, sensor, triage tool, triage, oximeter, pilot study, medical device

## Abstract

**Background:**

Carbon monoxide (CO) poisoning is an important cause of morbidity and mortality worldwide. Symptoms are mostly aspecific, making it hard to identify, and its diagnosis is usually made through blood gas analysis. However, the bulkiness of gas analyzers prevents them from being used at the scene of the incident, thereby leading to the unnecessary transport and admission of many patients. While multiple-wavelength pulse oximeters have been developed to discriminate carboxyhemoglobin (COHb) from oxyhemoglobin, their reliability is debatable, particularly in the hostile prehospital environment.

**Objective:**

The main objective of this pilot study was to assess whether the Avoximeter 4000, a transportable blood gas analyzer, could be considered for prehospital triage.

**Methods:**

This was a monocentric, prospective, pilot evaluation study. Blood samples were analyzed sequentially with 2 devices: the Avoximeter 4000 (experimental), which performs direct measurements on blood samples of about 50 µL by analyzing light absorption at 5 different wavelengths; and the ABL827 FLEX (control), which measures COHb levels through an optical system composed of a 128-wavelength spectrophotometer. The blood samples belonged to 2 different cohorts: the first (clinical cohort) was obtained in an emergency department and consisted of 68 samples drawn from patients admitted for reasons other than CO poisoning. These samples were used to determine whether the Avoximeter 4000 could properly exclude the diagnosis. The second (forensic) cohort was derived from the regional forensic center, which provided 12 samples from documented CO poisoning.

**Results:**

The mean COHb level in the clinical cohort was 1.7% (SD 1.8%; median 1.2%, IQR 0.7%-1.9%) with the ABL827 FLEX versus 3.5% (SD 2.3%; median 3.1%, IQR 2.2%-4.1%) with the Avoximeter 4000. Therefore, the Avoximeter 4000 overestimated COHb levels by a mean difference of 1.8% (95% CI 1.5%-2.1%). The consistency of COHb readings by the Avoximeter 4000 was excellent, with an intraclass correlation coefficient of 0.97 (95% CI 0.93-0.99) when the same blood sample was analyzed repeatedly. Using prespecified cutoffs (5% in nonsmokers and 10% in smokers), 3 patients (4%) had high COHb levels according to the Avoximeter 4000, while their values were within the normal range according to the ABL827 FLEX. Therefore, the specificity of the Avoximeter 4000 in this cohort was 95.6% (95% CI 87%-98.6%), and the overtriage rate would have been 4.4% (95% CI 1.4%-13%). Regarding the forensic samples, 10 of 12 (83%) samples were positive with both devices, while the 2 remaining samples were negative with both devices.

**Conclusions:**

The limited difference in COHb level measurements between the Avoximeter 4000 and the control device, which erred on the side of safety, and the relatively low overtriage rate warrant further exploration of this device as a prehospital triage tool.

## Introduction

Carbon monoxide (CO) poisoning is a leading cause of death, morbidity, and cost by poisoning worldwide [1]. It is classically encountered in winter, in enclosed spaces, and with any kind of combustion [2-4]. Symptoms are mostly aspecific, making CO poisoning difficult to identify. There can be gastrointestinal (nausea, vomiting, and abdominal pain), cardiopulmonary (dyspnea, chest pain, and syncope), and neurologic (headache, visual disturbance, ataxia, dizziness, delirium, coma, and seizures) manifestations that cannot be integrated into a specific toxidrome [2,3,5,6]. The diagnosis of CO poisoning is usually made by establishing high levels of carboxyhemoglobin (COHb) through blood gas analysis [7]. Medical management consists in enhancing CO clearance by exposure removal, supportive treatment, and above all, oxygenotherapy [3,8].

In most clinical settings, COHb levels are precisely and accurately determined by blood CO-oximetry through automated differential spectrophotometry; unfortunately, the bulkiness of regular blood gas analyzers prevents them from being used in the prehospital setting [7]. This drawback is noteworthy since fires—and more generally any incident potentially causing CO poisoning—can lead to multiple-casualty incidents [9-11]. In such situations, the stress caused by the fire and by the evacuation can generate aspecific symptoms akin to those of CO poisoning [12]. This can lead to the unnecessary transport and emergency department (ED) admission of many patients to rule out CO poisoning and can therefore potentially overload the regional health care system. A reliable tool for assessing COHb levels at the scene could therefore improve prehospital triage and maximize resources by preventing both pre- and in-hospital systems overload.

The current diagnostic tools adapted for prehospital use present many limitations that are partly conditioned by an imperative of transportability. Conventional pulse oximeters do not discriminate COHb from oxyhemoglobin (O_2_Hb) and display falsely reassuring oxygen saturation (SpO_2_) levels even in severe CO poisoning cases [13]. This is explained by the fact that most pulse oximeters only use 2 wavelengths to discriminate O_2_Hb from reduced hemoglobin (usually 660 and 930-940 nm) and that O_2_Hb and COHb present similar light absorption patterns at these wavelengths [14,15]. To overcome this limitation, multiple-wavelength pulse oximeters have been developed to better discriminate the light absorption patterns of O_2_Hb, COHb, reduced hemoglobin, and methemoglobin [14]. However, these devices are far from reliable even under ideal testing conditions [16-28]. Moreover, the reliability of many medical instruments in the prehospital setting, where hostile conditions such as lighting, excessive movements, and temperature can markedly influence monitoring, is debatable [29,30]. Indeed, there is little data regarding the reliability of pulse CO-oximetry in this context.

Since even regular pulse oximeters are prone to failures and inaccuracies in the prehospital setting, reliable and transportable devices using discrimination techniques akin to those of regular blood gas analyzers could prove useful to manage major incidents with potential CO poisoning victims [31-33]. To avoid unnecessary transports and admission, the principal objective of this pilot study was to assess whether the Avoximeter 4000 (Werfen), an on-site transportable blood gas analyzer, provided reliable COHb measurements in a population of adult patients a priori not intoxicated to CO, thus providing values near to the diagnostic thresholds used in clinical practice. The secondary objectives of this study were to determine if this analyzer provided accurate COHb measurements in forensic samples of proven CO poisoning cases as well as according to clinical thresholds commonly used in clinical practices.

## Methods

### Study Design and Setting

This was a monocentric, prospective pilot evaluation study carried out at the ED of the Geneva University Hospitals. We compared blood samples sequentially with 2 blood gas analyzers (one being tested and the other serving as a gold standard) in 2 different cohorts. Forensic samples were analyzed in August 2019, and samples were collected from live patients between June and August 2022. The results were then recorded immediately without any personal authentication data (apart from age and gender) in a digital Excel sheet (Microsoft Excel, version 2013; Microsoft Corporation).

### Ethical Considerations

Consent was waived by the regional ethics committee (Commission Cantonale d’Ethique de la Recherche CCER project-ID 2019-01048) since only already drawn blood samples were used for the purpose of this study. Since only residuals from blood samples already drawn for clinical purposes were used, the ethics committee waived the need for consent, thereby also preventing any kind of compensation. In addition, there was no contact between the patients from whom the blood was drawn and the investigators. Patient confidentiality was guaranteed by the fact that no identifying information was ever even asked for, let alone recorded.

### Blood Gas Analyzers

The ABL827 FLEX blood gas analyzer (Radiometer) was used as the gold standard. This blood gas, oximetry, electrolyte, and metabolite analyzer is a complex modular device composed of several units, one of which is dedicated to oximetry studies. This unit measures COHb levels through an optical system composed of a 128-wavelength spectrophotometer. This optical system, which was already used in prior models, was validated using gas chromatography. The ABL827 FLEX is routinely used in the ED, and internal quality controls are carried out twice a day. In addition, 4 external quality controls are carried out every year.

The Avoximeter 4000 is a blood oximeter that performs hemoglobin, O_2_Hb, COHb, and methemoglobin direct measurements on blood samples of about 50 µL by analyzing light absorption at 5 different wavelengths. The drawn blood is injected into a single-use cuvette, which is inserted into the device. Results are delivered in 7 to 10 seconds. This device can easily be transported by virtue of its reasonable size (20.3 cm×25.4 cm×9.5 cm) and weight (1.8 kg). The accuracy and precision claimed by the manufacturer are ±2% and 1%, respectively.

An Avoximeter 4000 as well as 100 cuvettes were provided free of charge by Axon Lab AG for the purpose of this study. Quality controls were performed according to the manufacturer’s instructions throughout the study period.

### Blood Samples

Blood samples were collected from a cohort of 68 adult patients aged 18 years or older admitted to the ED of the Geneva University Hospitals between June and August 2022. None of these patients was admitted for CO poisoning. There were no exclusion criteria. The samples were collected from patients who required blood gas analysis through the usual ABL827 FLEX analyzer for their own clinical management. Residuals are almost always present in the syringes, and 50 µL of the remaining blood samples were therefore immediately used to perform the analysis on the Avoximeter 4000 device. The majority were of venous origin (n=55, 81%), and the rest were arterial (n=13, 19%). These samples were meant to evaluate if the tested CO-oximeter could properly exclude the diagnosis.

The second cohort was derived from the regional forensic center (Centre Universitaire Romand de Médecine Légale, the French-speaking Swiss university center of forensic medicine), which provided 12 samples from documented CO poisoning that were analyzed by the 2 methods on August 8, 2019; this group was meant to assess the ability of the Avoximeter 4000 to identify CO poisoning cases.

### Data Collection and Outcomes

All specimens were sequentially analyzed with the ED blood gas analyzer (ABL827 FLEX) and the point-of-care CO-oximeter (Avoximeter 4000) to compare COHb levels. A subset of samples from the cohort of ED patients (n=8, 11%) were analyzed several times with the Avoximeter 4000 to estimate the intraindividual consistency of the device.

The primary outcome was the difference in the measure of COHb (in percentage) between the 2 devices. Secondary outcomes were the reliability of the measure with the Avoximeter 4000 and the overtriage rate of the Avoximeter 4000 when applying the diagnostic thresholds used in common clinical practice (<5% for nonsmokers and <10% for smokers) [8,24]. In the forensic cohort, the smoking status was unknown, and a diagnostic threshold of 5% was therefore applied to all samples.

### Data Analysis

Stata (version 17.0; StataCorp LLC) was used for statistical analysis. First, a quantitative analysis with the Bland-Altman diagrams as a measure of agreement was performed. Then, positive and negative results were defined according to the aforementioned diagnostic thresholds. This allowed to determine the overtriage rate and the Avoximeter 4000’s specificity. To assess the reliability of the Avoximeter 4000 through repeated measurements, an intraclass correlation coefficient was computed.

## Results

### Patients

The median age of the patients in the live sample cohort (n=68) was 64.5 (IQR 42.5-82) years. There were 22 (32%) active smokers. These patients were admitted because of neurologic (n=20, 29%), cardiorespiratory (n=19, 28%), gastrointestinal (n=9, 13%), and traumatological (n=8, 12%) problems.

### Primary Outcome

**Figure 1:**
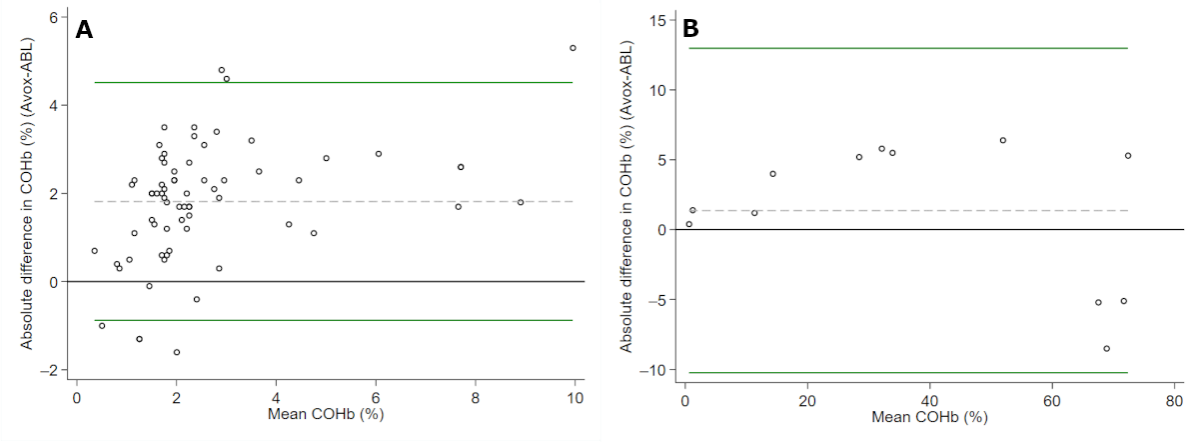
Bland-Altman plots featuring differences in COHb between the Avoximeter 4000 and the ABL827 Flex (Avox-ABL) blood gas analyzers according to mean values. (A) Live samples and (B) forensic specimens. COHb: carboxyhemoglobin.

The mean COHb level in the clinical cohort was 1.7% (SD 1.8%; median 1.2%, IQR 0.7%-1.9%) with the ABL827 FLEX versus 3.5% (SD 2.3%; median 3.1%, IQR 2.2%-4.1%) with the Avoximeter 4000. Therefore, the Avoximeter 4000 overestimated COHb levels by a mean difference of 1.8% (95% CI 1.5%-2.1%). The agreement between the measures provided by these devices is shown in Figure 1.

**Figure 2:**
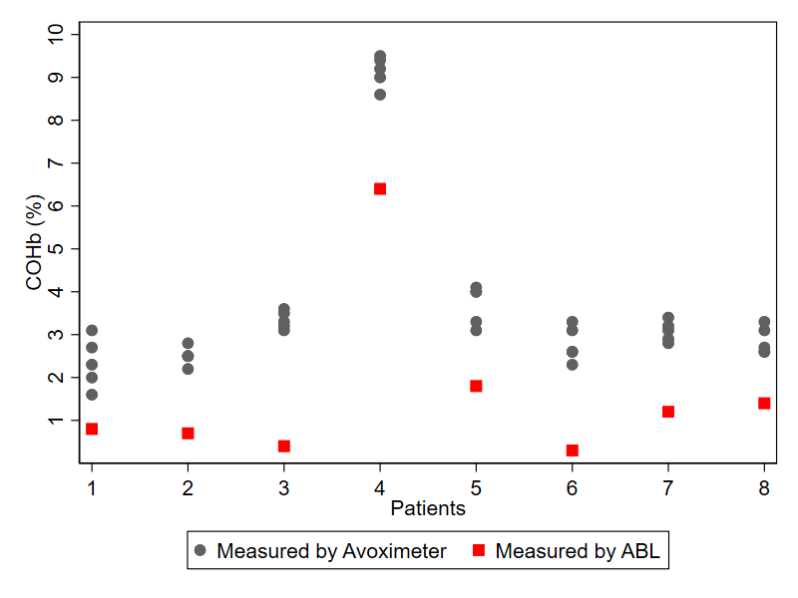
Correlation between different measurements carried out on the same 8 blood samples. COHb: carboxyhemoglobin.

The consistency of COHb readings by the Avoximeter 4000 is presented in Figure 2. The correlation of measurements made on the same individual was excellent, with intraclass correlation coefficient of 0.97 (95% CI 0.93-0.99).

### Secondary Outcomes

#### Clinical Cohort

Using the prespecified cutoffs (5% in nonsmokers and 10% in smokers), 3 patients (4%) had high COHb levels according to the Avoximeter 4000, while their values were within the normal range according to the ABL827 FLEX. Therefore, the specificity of the Avoximeter 4000 in this cohort was 95.6% (95% CI 87%-98.6%), and the overtriage rate would have been 4.4% (95% CI 1.4%-13%). The sensitivity cannot be estimated in this clinical cohort due to the absence of a true positive test for CO poisoning.

#### Forensic Cohort

Regarding the forensic samples, 10 of 12 (83.3%) samples were positive with both devices, while the 2 remaining samples were negative with both devices. Theoretically, this would represent a sensitivity of 100%, but these results should be interpreted with great caution given the limited sample size and the origin of these samples.

## Discussion

### Principal Findings

In this study, the Avoximeter 4000, an on-site transportable blood gas analyzer, provided reliable COHb measurements in a population of adult patients admitted to an ED for reasons other than CO poisoning.

Overall, the Avoximeter 4000 generally led to a mild overestimation of COHb levels. The difference found between COHb values was limited and erring on the side of safety, which is undeniably preferable in this setting. In addition, the rate of overtriage, which was less than 5%, is probably acceptable for most prehospital systems. Indeed, overtriage is probably higher if triage is only based on potential CO poisoning symptoms given their aspecific nature.

The difference in COHb levels was higher in forensic specimens. This could be either due to the very nature of these specimens, to less accurate measurements with higher COHb concentrations, or both. Regardless of the reasons underlying this discrepancy, the differences reported in this study would not have altered the clinical management of such patients since there was a good agreement according to the diagnostic thresholds used in practice.

The main limitation of this study is the lack of CO poisoning samples from living patients, which made it impossible to estimate the sensitivity of this device in this population. This was however mitigated by the inclusion of the forensic samples.

Another limitation is the fact that the assessment was carried out in an ED rather than in the field. However, the bulk of the device and the quick acquisition of the results once samples have been collected all support further evaluation in the prehospital environment.

The encouraging results reported here support further assessment of the Avoximeter 4000 as a potential prehospital triage tool in case of CO poisoning. It could be especially useful in a large-scale event to avoid overloading hospital systems without compromising patient safety by virtue of a reasonable false negative rate. This could also limit costs and save resources by turning what is currently a hospital triage logic into a prehospital one.

Thus, this pilot study lays the ground for a full-fledged prehospital study. Such a study will certainly take time since fires are unpredictable events, and researchers will need to be able to respond round the clock. The potential benefits are undoubtedly worth the effort since increasing on-site triage efficiency and avoiding unnecessary transports to the hospital could help decrease ED overcrowding, which represents a challenging and critical public health issue.

### Conclusions

This pilot assessment showed a good correlation between the Avoximeter 4000 and the gold standard blood gas analyzer regarding COHb levels. By somewhat erring on the safe side, the Avoximeter 4000 would have led to a 4.4% overtriage rate in a real setting. This portable CO-oximeter should now be tested in the field to assess its actual yield in case of a major incident with potential CO poisoning.

## Abbreviations

CO: carbon monoxide
COHb: carboxyhemoglobin
ED: emergency department
O2Hb: oxyhemoglobin
SpO2: oxygen saturation

## Data Availability

The data sets generated and analyzed during this study are available from the corresponding author on reasonable request.
